# Antidepressant Interactions with the NMDA NR1-1b Subunit

**DOI:** 10.1155/2008/474205

**Published:** 2008-06-05

**Authors:** Richard Raabe, Lisa Gentile

**Affiliations:** Department of Chemistry, University of Richmond, 28 Westhampton Way, Richmond, VA 23173, USA

## Abstract

The targets for tricyclic antidepressants (TCAs), selective serotonin reuptake inhibitors (SSRIs), and selective norepinephrine reuptake inhibitors (SNRIs) are known to be the serotonin and norepinephrine transport (reuptake) proteins which are embedded in presynaptic nerve terminals and function to bring these neurotransmitters from the synaptic cleft back into the presynaptic neuron. Using a combination of intrinsic and extrinsic fluorescence quenching, Stern-Volmer analysis, and protease protection assays, we have shown that therapeutics from each of these three classes of antidepressants bind to the extracellular S1S2 domain of the NR1-1b subunit of the NMDA receptor. These results are in agreement with recent work from our lab demonstrating the interaction of antidepressants with the S1S2 domain of the GluR2 subunit of the AMPA receptor, another member of the ionotropic glutamate receptor subfamily, as well as work from other labs, and continue the discussion of the involvement of ionotropic glutamate receptors in depression.

## 1. Introduction

Ionotropic glutamate receptors (iGluRs) are a family of ligand-gated ion channels located on the postsynaptic neural membrane which open in response to extracellular binding of the neurotransmitter glutamate. These receptors play an important role in memory, learning, development, and neu-ral plasticity. As such, their misregulation has been implicated in a number of disease states such as the ischaemic stroke cascade, schizophrenia, epilepsy, and Alzheimer's, Huntington's, and Parkinson's Disease.

**Figure 1:**
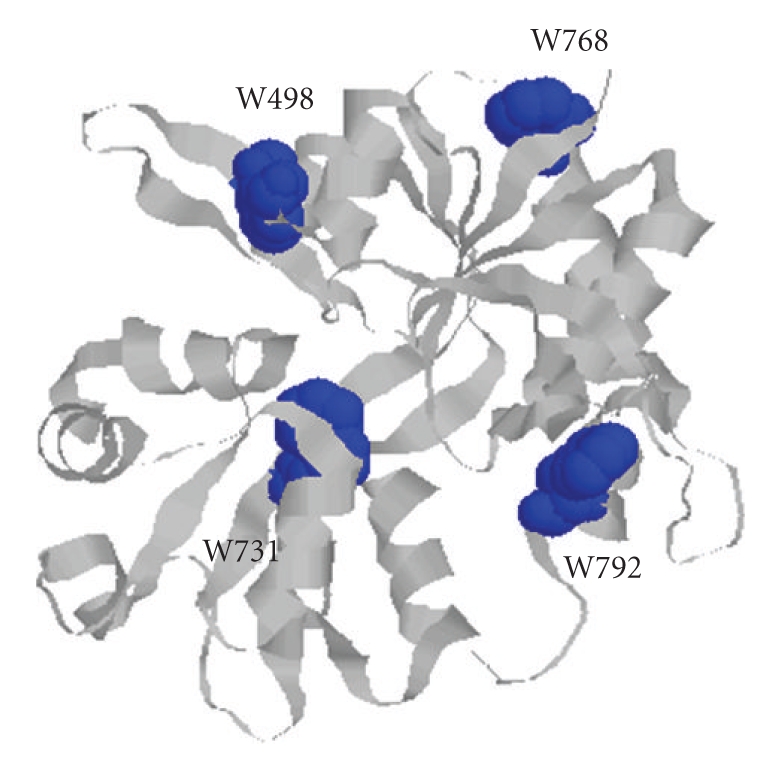
Crystal structure of the NR1-1b S1S2 domain (PDB code 1PB7) [4] with each of the four tryptophans in blue.

The functional unit of each member of the iGluRfamily (NMDA, AMPA, and kainate receptors) is a tetramer; homotetramer for AMPA and kainate receptors and heterotetramer, composed of NR1 (one of eight alternatively spliced versions) and NR2 subunits (one of four versions encoded by four different genes) for NMDA receptors. Subunits from any of these family members are modular in nature, containing seven domains: an amino terminal domain (ATD) important for the interaction between subunits, a nonsequential extracellular S1S2 neurotransmitter binding do main, three membrane spanning domains, a re-entrant loop which forms the pore of the ion channel when the tetramer is assembled, and an intracellular C-terminal tail. Soluble extracellular GluR2 (AMPA) and NR1-1b (NMDA) S1S2 domains have been constructed by eliminating all three transmembrane spanning regions plus the re-entrant loop and linking the S1 and S2 domains by two amino acids (GT) [1–4]. These S1S2 domains have been shown to possess near native binding affinities for both agonists and antagonists and their structures have been studied by X-ray crystallography (Figure 1) [1–4].

In the past twenty years, there has been an accumulation of data suggesting that iGluRs are also involved in the mechanism of action of antidepressants. This is a de-parture from the idea that antidepressants function solelyby either inhibiting monoamine oxidase (MAOIs) or the serotonin or norepinephrine transport proteins (TCAs, SSRIs, or SNRIs). Data linking iGluRs to antidepressant activities include experiments demonstrating direct binding of tricyclic antidepressants to the extracellular S1S2 region of AMPA receptors [5, 6], open channel block of NMDA receptors by antidepressants [7, 8], and altered expression and phosphorylation of AMPA receptors by antidepressants [9, 10]. In addition, AMPA receptor potentiators and NMDA receptor antagonists have been shown to have antidepressant-like effects [9, 11–13]. This data has led to the suggestion that increased activity of AMPA receptors leads to an increase in the expression of brain derived neurotrophic factor (BDNF), which in turn promotes neurogenesis in the hippocampus, leading to antidepressant activities [9].

**Figure 2:**
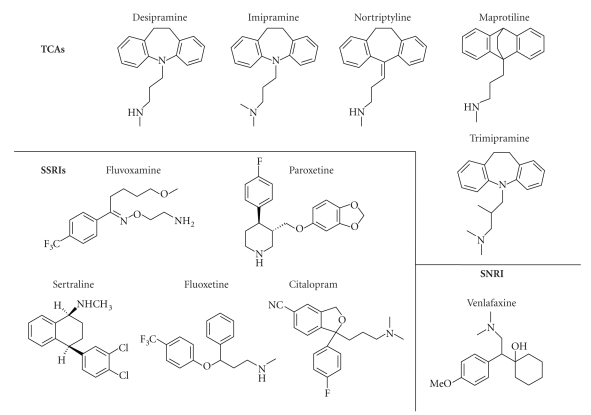
The TCAs, SSRIs, and SNRIs above, with the exception of citalopram, were found to bind to the NMDA NR1-1b S1S2 domain.

The intrinsic and extrinsic fluorescence studies presented here add to the body of knowledge suggesting a role for NMDA receptors in antidepressant activities. Specifically, they demonstrate that five tricyclic antidepressants (TCAs) (desipramine, trimipramine, maprotiline, nortriptyline, and imipramine), four selective serotonin reuptake inhibitors (SSRIs) (fluvoxamine, paroxetine, sertraline, and fluoxetine), and one selective norepinephrine reuptake inhibitor (SNRI) (venlafaxine), see Figure 2, bind to the S1S2 domain of the NR1-1b subunit of the NMDA receptor. The only antidepressant that showed no binding in any of our studies, at concentrations as high as 5.21 mM, was the SSRI citalopram.

## 2. Experimental Procedures

### 2.1. Protein Expression and Purification of the NMDA NR1-1b S1S2 Domain

The cloning plasmid for the NMDA NR1-1b S1S2 domain, provided by Eric Gouaux (Oregon Health and ScienceUniversity), was chemically transformed into *E. coli* Origami B(DE3) cells. The expression system was designed as follows: (His)_8_-TSG-LVPRG(thrombin cut site)-S1(394-544)-GT-S2(663-800). Expression and purification of NMDA NR1-1b S1S2 protein were performed as in Furukawa and Gouaux [4].

### 2.2. Intrinsic Fluorescence

Intrinsic fluorescence studies were performed in a Cary Eclipse fluorometer at 25°C. All emission scans were acquired from 300 to 400 nm upon excitation at 290 nm, with 10 nm excitation and emission slit widths in a 10 mm-path-length cell. Excitation was performed at 290 nm to minimize interference from both tyrosine residues in the NMDA NR1-1b S1S2 domain as well as the aromatic antidepressants being studied. Before data acquisition, samples were incubated for thirty seconds in the sample holder at 25°C. Analysis of the buffer emission signal showed that it contributed less than half a percent to the fluorescence intensity of the NR1-1b S1S2 domain. Thus, data was reported without a buffer subtraction.

Protein samples were at a final concentration of 5.29 *μ*M in buffer 3 (10 mM MES, pH 6.5, 25 mM NaCl, 1 mM glycine). All antidepressants were solubilized in 100% methanol to the highest attainable concentrations. To account for any effects methanol had on fluorescence intensity, protein spectra with methanol were collected, allowing direct comparison between samples with and without antidepressant. Each data set consisted of at least three independent emission spectra of identically prepared samples. The data is reported with error determined at the 95% confidence level.

### 2.3. Intrinsic Quenching Studies

The fluorescence intensity of the NR1-1b S1S2 domain was determined in the presence and absence of eachantidepressant with the appropriate blank subtraction. The final concentrations of each TCA-desipramine, imipramine, trimipramine, nortriptyline, and maprotiline were 136.9 *μ*M, 136.9 *μ*M, 136.9 *μ*M, 2.08 mM, and 3.18 mM, respectively. The final concentrations of each SSRI-paroxetine, citalopram, and sertraline were 3.75 mM, 1.88 *μ*M, and 1.88 mM, respectively. For all samples, the final concentration of methanol was kept at 1.8%.

### 2.4. *K*_*d*_ Determination

Antidepressants were added to final concentrations as follows: nortriptyline: 1.24–3.61 mM, imipramine or desipramine: 8–570 *μ*M, trimipramine: 130–570 *μ*M, sertraline: 0.74–1.84 mM, fluvoxamine: 0.74–2.37 mM. A double reciprocal plot was used to determine *K_d_*, which derives from the Benesi-Hildebrand relationship, provided there is a 1 : 1 binding stoichiometry. The equation is: 1/Δ*F* = 1/((*F*_*∞*_ − *F*_*o*_)·*K*_*d*_[*L*]) + 1/(*F*_*∞*_ − *F*_*o*_), where *F_o_* is the fluorescence intensity in the absence of ligand, *F*_*∞*_ is the fluorescence intensity with saturating concentration of ligand, *F* is thefluorescence intensity at some particular [ligand], Δ*F* is (*F* − *F*_*o*_), and *K_d_* is the dissociation constant. When 1/Δ*F* is plotted against 1/[*L*], a linear relationship exists. The intercept on the *x*-axis is − *K*_*d*_. [14]. Note, this method to determine *K_d_* is based on the formation of ground state NMDA NR1-1b S1S2 domain: antidepressant complexes, and could be altered by dynamic quenching.

### 2.5. Stern-Volmer Analysis

Acrylamide (Sigma) ranging in final concentration from 16.3 to 113.9 mM was added to 5.29 *μ*M NR1-1b S1S2 premixed with antidepressant (at final concentrations as above for intrinsic quenching studies, with the exception of sertraline which was at 0.94 mM). Plots of (*F*_0_/*F*) −1 versus acrylamide concentration were constructed.

### 2.6. Extrinsic Fluorescence

Extrinsic fluorescence studies were performed in a Cary Ec-lipse fluorometer at 25°C. All emission scans were acquired from 400 to 600 nm after excitation at 380 nm with 20 nm slit widths for emission and excitation in a 10 mm-path-length cell. Before data acquisition, samples were incubated for thirty seconds in the sample holder at 25°C. Blanks were collected both for the antidepressants and the NR1-1b S1S2 domain. Three emission spectra of identicallyprepared samples were averaged for each plot. 8-Anilino-1-naphthalenesulfonic acid (ANS, TCI America), in a 1 : 1 molar ratio, was added to 5.29 *μ*M of the NR1-1b S1S2 domain premixed with antidepressant (at final concentrations as above for intrinsic quenching studies in addition to the SSRIs fluoxetine (8.62 *μ*M) and fluvoxamine (2.81 mM) and the SNRI venlafaxine (29.07 *μ*M)). As with all intrinsic assays, total methanol concentration was kept at 1.8%. Plots of intensity versus wavelength were constructed from averaged emission spectra.

### 2.7. Protease Protection

Antidepressants (at final concentrations as above for the extrinsic assays, with the exception of sertraline which was added to a final concentration of 940 *μ*M) were added to 60 *μ*L aliquots of the NR1-1b S1S2 domain at a final concentration of 5.29 *μ*M. The NR1-1b S1S2 domain/antidepressant complexes were allowed to incubate on ice for 5 minutes before trypsin (Sigma: 13500 U/mg) was added to a final concentration of 5.29 *μ*M, yielding a 1 : 1 molar ratio of protein to trypsin. The reactions were then incubated at room temperature for 45 minutes. Digestion was stopped by the addition of sample loading dye, DTT (50 mM), and incubation at 100°C for 10 minutes. Each reaction was visualized by 12% SDS-PAGE.

## 3. Results and Discussion

### 3.1. Intrinsic Tryptophan Fluorescence Demonstrates Binding of 5 TCAs and 3 SSRIs to the NMDA NR1-1b S1S2 Domain

**Figure 3:**
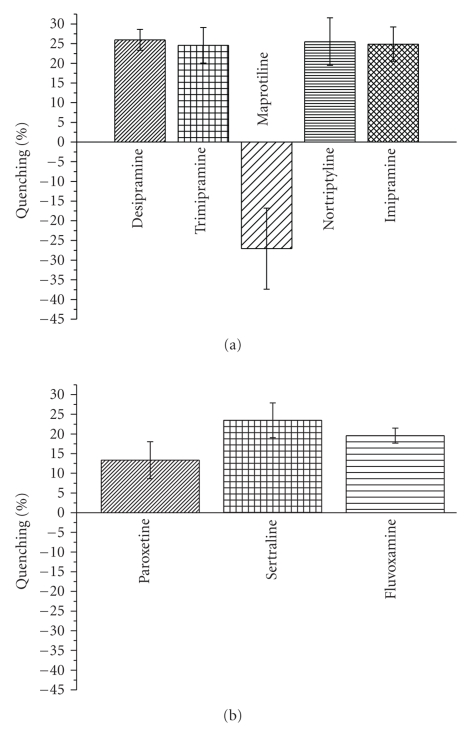
Intrinsic fluorescence quenching of the NR1-1b S1S2 domain with (a) TCAs and (b) SSRIs. To 5.29 *μ*M NR1-1b S1S2 in buffer 3 was added quenchers in the following final concentrations: (a) TCAs-desipramine, trimipramine, or imipramine: 137 *μ*M, maprotiline: 3.18 mM, nortriptyline: 2.08 mM; (b) SSRIs-paroxetine: 3.75 mM, sertraline: 1.88 mM, fluvoxamine: 2.81 mM.

The environment of the four tryptophan residues in the NMDA NR1-1b S1S2 domain, W498, W731, W768, W792, can be monitored by fluorescence spectroscopy. Uponexcitation, the emission maximum (*λ*_max _) and fluorescence intensity of a tryptophan residue depends upon the polarity of its environment. By comparing the emission spectrum (*λ*_max _ and fluorescence intensity) of the apo NMDA NR1-1b S1S2 domain to antidepressant bound complexes, it is possible to determine if the antidepressant has bound to the S1S2 domain and caused a change in the environment of the NR1-1b S1S2 tryptophan residues. An increase in the polarity of the environment surrounding a tryptophan causes an increase in the *λ*_max _ of emission and a decrease in the fluorescence intensity. The data in Figure 3(a), which monitors the intensity of the tryptophan emission from the NMDA NR1-1b S1S2 domain shows binding of the TCAs desipramine (*K_d_*: 730 ± 150 *μ*M; for *K_d_* data see supplemental Figures 1S and 2S), trimipramine (*K_d_*: 1.09 ± 0.24 mM), maprotiline, nortriptyline (*K_d_*: 7.36 ± 2.06 mM), and imipramine (*K_d_*: 830 ± 150 *μ*M). The data in Figure 3(b) demonstrates the binding of the SSRIs paroxetine, sertraline (K*_d_*: 240 ± 90 *μ*M), and fluvoxamine (*K_d_*: 6.54 ± 3.48 mM). These intrinsic fluorescence studies are complicated by the fact that paroxetine, sertraline, citalopram, and venlafaxine, the former two that showed evidence of binding to the NR1-1b S1S2 domain, and the latter two that did not, all had relatively high intrinsic fluorescence signals in the emission range of tryptophan. To compensate for this, lower drug concentrations had to be used which reduced the range of workable concentrations, proving problematic for *K_d_* determination. Low drug solubility in methanol (one of the only organics compatible with a folded S1S2 domain) further complicated *K_d_* determination for maprotiline and sertraline.

Based on this intrinsic fluorescence quenching data, the only antidepressant which either binds in a unique location on the S1S2 domain or causes a unique conformational change upon binding is maprotiline, the binding of which increases, rather than quenches, the tryptophan fluorescence emission. The unique mode of binding of maprotiline is further supported by the shift in the maximum wavelength of emission of the maprotiline bound S1S2 complex, from 326.24 ± 1.54 nm (apo) to 322.04 ± 1.83 nm (bound), Table 1. This decrease in *λ*_max _ and increase in fluorescence emission upon binding of maprotiline are consistent with the tryptophan residues being in a more nonpolar environment upon complex formation, either due to direct contact with maprotiline or to a conformational change upon maprotiline binding that places them in a more nonpolar portion of the protein.

The only other ligand which caused a statisticallysignificant shift in *λ*_max _ of the S1S2 domain is sertraline, which upon complex formation is shifted to 329.07 ± 0 nm (Table 1). This increase in *λ*_max _ and decrease in fluorescence emission upon binding of sertraline, the tightest binding antidepressant used in these studies, are consistent with the tryptophan residues being in a more polar environment upon complex formation, either due to direct contact with sertraline or to a conformational change upon sertraline binding that places them in a more polar or solvent exposed portion of the protein.

### 3.2. Extrinsic ANS Fluorescence Confirms the Binding of 4 TCAs and 3 SSRIs and Demonstrates Binding of Fluoxetine (SSRI) and Venlafaxine (SNRI) to the NMDA NR1-1b S2S2 Domain

**Figure 4:**
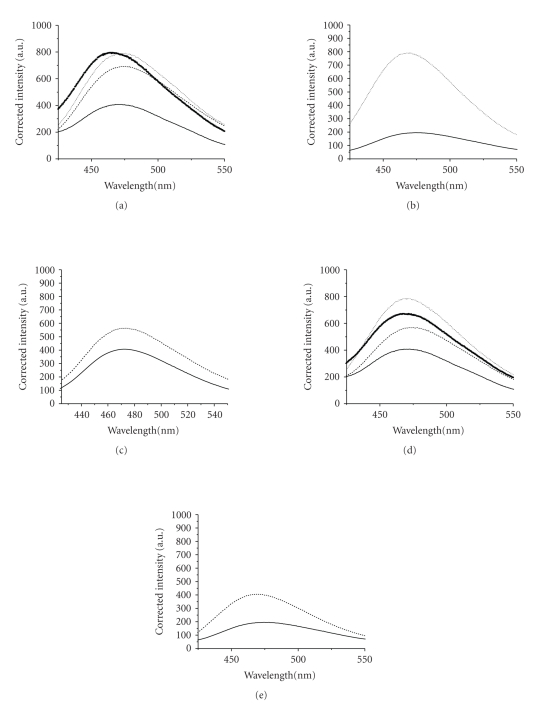
ANS binding. To 5.29 *μ*M NR1-1b S1S2 in buffer 3 was added ANS in a 1 : 1 molar ratio along with a TCA, SSRI, or SNRI in the following final concentrations: (a) TCAs-desipramine (- - -): 137 *μ*M, trimipramine (⋯): 137 *μ*M, maprotiline (▪): 3.18 mM; (b) TCAs-nortriptyline (⋯): 2.08 mM; (c) SNRI-venlafaxine (- - -): 2.76 mM; (d) SSRI-fluvoxamine (- - -): 2.81 mM, paroxetine (⋯): 3.75 mM, sertraline (▪): 1.88 mM; (e) SSRI-fluoxetine (- - -): 862 *μ*M. In all figures the solid line is the extrinsic fluorescence of ANS:NR1-1b S1S2. In all figures, intensities were corrected by subtracting the drug and ANS blank.

If an antidepressant bound to the S1S2 domain of the NR1-1b subunit of the NMDA receptor, but did not change the polarity surrounding the tryptophans or changed them in such a way that the results canceled each other out, it would not be possible to determine binding from the intrinsic fluorescence studies described above. To address this, the extrinsic fluor ANS was used to probe antidepressant binding. Upon binding to hydrophobic patches on the surface of proteins, the *λ*_max _ of ANS decreases and its emission intensity increases. As can be seen in Figures 4(a) and 4(b), the emission spectrum of ANS bound to NR1-1b S1S2 complexes of desipramine, trimipramine, maprotiline, or nortriptyline was significantly different from ANS bound to the apo S1S2 domain, confirming the binding of these antidepressants. Again, the uniqueness of maprotiline binding is confirmed by the shift in *λ*_max _ of the complex. No data is presented for the ANS bound NR1-1b S1S2-imipramine complex. In this case, a strong, shifted fluorescence signal from the ANS, imipramine, buffer blank suggested a direct binding of ANS to imipramine.

The three SSRIs that showed binding by intrinsic fluorescence quenching, sertraline, paroxetine, and fluvoxamine,all had spectra that were significantly different from the apo spectrum, confirming their binding (Figure 4(d)). In addition, ANS emission spectra confirmed the binding of both the SNRI venlafaxine (Figure 4(c)) and the SSRI fluoxetine (Figure 4(e)), neither of which showed binding in the intrinsic fluorescence assays. Citalopram is the only antidepressant studied which showed no evidence of bindingin either the intrinsic or extrinsic assays at concentrations as high as 5.21 mM.

### 3.3. Stern-Volmer Quenching Analysis Differentiates Conformational Changes Observed Upon Antidepressant Binding

To probe the conformational change elicited upon binding of each of the antidepressants, a Stern-Volmer analysis was performed. In these experiments, the ability of an external molecule, in this case acrylamide, to quench tryptophan fluorescence is monitored in both the apo NR1-1b S1S2 and in the antidepressant bound NR1-1b S1S2 domains. The Stern-Volmer constant, *K*_sv_, defined as the product of the bimolecular collisional constant, *k_q_*, and the lifetime of the tryptophan residues in the absence of quencher, **τ*_0_*, is measured. The bimolecular collisional constant is a measure of the accessibility of acrylamide to the tryptophans with an increase in *k_q_* equating to an increase in accessibility to the fluorophore. If it is assumed that the lifetime of the tryptophans do not change upon antidepressant binding [15–17], then changes in *K*_sv_ are due to changes in *k_q_* and therefore reflect the change in accessibility of the tryptophan residues to acrylamide upon antidepressant binding. The data in Table 2 shows that upon binding to any of the antidepressants, the accessibility of the tryptophan residues in the S1S2 domain to acrylamide increased when compared to the apo protein. Within the TCAclass of antidepressants, the conformational change upon imipramine/trimipramine bindingappears distinct from that of desipramine/nortriptyline binding, whichappears distinct from maprotiline binding. In these assays, the binding of imipramine could not be distinguished from trimipramine, and the binding of desipramine could not be distinguished from nortriptyline. Within the SSRI class of antidepressants, all conformational changes elicited upon binding to the NR1-1b S1S2 domain were found to alter the accessibility of the tryptophan residues in statistically significant ways.

### 3.4. Protease Protection Assays Differentiate Conformational Changes of Nortriptyline and Desipramine Binding to the NMDA NR1-1b Domain

**Figure 5:**
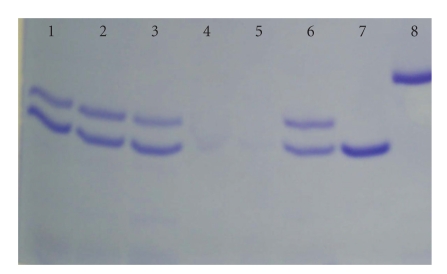
Protease protection assay of NR1-1b S1S2:TCA complexes. To 5.29 *μ*M NR1-1b S1S2 in buffer 3 was added the final quenching concentrations of TCAs prior to addition of a 1 : 1 molar ratio of trypsin. The results of the trypsin cuts were visualized by 12% SDS PAGE. Lanes were loaded as follows, (1): desipramine-bound, (2): imipramine-bound, (3): apo NR1-1b S1S2, (4): nortriptyline-bound, (5): maprotiline-bound, (6): trimipramine-bound, (7): trypsin, (8): uncut NR1-1b S1S2.

To differentiate the binding of desipramine and nortriptyline as well as trimipramine and imipramine to the S1S2 domain of the NR1-1b subunit, a limited trypsin digest was performed on the antidepressant bound complexes. Figure 5 shows that this method was unsuccessful in differentiating the conformational changes associated with imipramine (lane 2) and trimipramine (lane 6) binding. However, it was successful in differentiating the conformational changes associated with nortriptyline (lane 4) and desipramine (lane 1) binding with the conformational change associated with nortriptyline affording much less protection to proteolysis than the desipramine bound complex or the apo S1S2 do-main.

### 3.5. Summary

A combination of intrinsic and extrinsic fluorescence binding studies, Stern-Volmer analysis, and protease protection assays provides evidence for the binding of five TCAs, four SSRIs, and one SNRI to the NMDA NR1-1b S1S2 domain. Data from the SSRI class of antidepressants supports the hypothesis that the conformational changes elicited upon binding to the S1S2 domain are unique. Within the TCA class of antidepressants, the conformational changes of the S1S2 domain upon binding to each drug also appear to be unique, with the exception of binding to imipramine and trimipramine. Structurally, the difference between imipramine and trimipramine (Figure 2) is the branched aliphatic side chain present in trimipramine. While it is possible that this additional steric hindrance in trimipramine causes it either to bind to a unique site or in a unique way to the same site within the S1S2 domain, it is also possible that these two drugs have very similar binding to the S1S2 domain of the NR1-1b subunit. None of the studies performed provide evidence of binding of this iGluR domain to the SSRI citalopram. Perhaps the presence of the nitrile group, present in none of the other antidepressants studied, prevents binding to the NR1-1b S1S2 domain.

Based upon the ways in which iGluRs have been proposed to interact with antidepressants, this direct binding to the extracellular S1S2 domain of the NMDA NR1-1b subunit may fit into the previously observed category of NMDA antagonists having antidepressant activities [9, 11–13]. As the TCAs have been shown to produce open channel block of the NMDA receptors, however, differentiating antagonist effects from external S1S2 binding and open channel block will be difficult [7, 8]. As is the case for open channel block, for direct binding of antidepressants to the NMDA NR1-1b S1S2 domain to play a major role in the mode of action of these drugs, the differences in their binding affinities (*K_d_* range 0.24–7.36 mM), and typical therapeutic plasma concentrations (0.17–1 *μ*M), will need to be resolved [7, 8, 18, 19]. Part of this solution may lie in the fact that while these isolated iGluR S1S2 domains have been shown to possess near native binding affinities for both agonists and antagonists in vitro, their in vivo interactions with antidepressants could be quite different [1–3, 20–22].

## Figures and Tables

**Table 1:** *λ*_max _ of the NR1-1b S1S2 apo domain and of the NR1-1b drug bound complexes.

Antidepressant	*λ*_max _ (nm)	Standard deviation (nm)
Desipramine	326.77	1.90
Nortriptyline	327.8	0.950
Imipramine	328.04	1.38
Trimipramine	327.27	2.19
Maprotiline	322.04	1.83
Fluvoxamine	328.57	1.91
Paroxetine	328.3	0.960
Sertraline	329.07	0
Apo	326.24	1.54

**Table 2:** *K*_sv_ values for the NMDA NR1-1b S1S2 apo domain and for the NR1-1b antidepressant complexes.

Antidepressant (family)	*K*_sv_ (*μ*M^-1^)	Error in *K*_sv_ (*μ*M^−1^)
Desipramine (TCA)	2.67	0.10
Nortriptyline (TCA)	2.56	0.17
Imipramine (TCA)	3.15	0.06
Trimipramine (TCA)	3.02	0.21
Maprotiline (TCA)	5.92	0.47
Sertraline (SSRI)	2.23	0.10
Paroxetine (SSRI)	3.06	0.13
Fluvoxamine (SSRI)	3.77	0.07
Apo	1.61	0.11

## References

[1] Armstrong N, Gouaux E (2000). Mechanisms for activation and antagonism of an AMPA-sensitive glutamate receptor: crystal structures of the GluR2 ligand binding core. *Neuron*.

[2] Chen G-Q, Sun YU, Jin R, Gouaux E (1998). Probing the ligand binding domain of the GluR2 receptor by proteolysis and deletion mutagenesis defines domain boundaries and yields a crystallizable construct. *Protein Science*.

[3] Chen G-Q, Gouaux E (1997). Overexpression of a glutamate receptor (GluR2) ligand binding domain in *Escherichia coli*: application of a novel protein folding screen. *Proceedings of the National Academy of Sciences of the United States of America*.

[4] Furukawa H, Gouaux E (2003). Mechanisms of activation, inhibition and specificity: crystal structures of the NMDA receptor NR1 ligand-binding core. *The EMBO Journal*.

[5] Stoll L, Seguin S, Gentile L (2007). Tricyclic antidepressants, but not the selective serotonin reuptake inhibitor fluoxetine, bind to the S1S2 domain of AMPA receptors. *Archives of Biochemistry and Biophysics*.

[6] Stoll L, Gentile L (2005). Linking tricyclic antidepressants to ionotropic glutamate receptors. *Biochemical and Biophysical Research Communications*.

[7] Reynolds IJ, Miller RJ (1988). Tricyclic antidepressants block *N*-methyl-D-aspartate receptors: similarities to the action of zinc. *British Journal of Pharmacology*.

[8] Sernagor E, Kuhn D, Vyklicky L, Mayer ML (1989). Open channel block of NMDA receptor responses evoked by tricyclic antidepressants. *Neuron*.

[9] Bleakman D, Alt A, Witkin JM (2007). AMPA receptors in the therapeutic management of depression. *CNS & Neurological Disorders-Drug Targets*.

[10] Svenningsson P, Tzavara ET, Witkin JM, Fienberg AA, Nomikos GG, Greengard P (2002). Involvement of striatal and extrastriatal DARPP-32 in biochemical and behavioral effects of fluoxetine (Prozac). *Proceedings of the National Academy of Sciences of the United States of America*.

[11] Quirk JC, Nisenbaum ES (2002). LY404187: a novel positive allosteric modulator of AMPA receptors. *CNS Drug Reviews*.

[12] Jin R, Clark S, Weeks AM, Dudman JT, Gouaux E, Partin KM (2005). Mechanism of positive allosteric modulators acting on AMPA receptors. *Journal of Neuroscience*.

[13] Sanacora G, Gueorguieva R, Epperson CN (2004). Subtype-specific alterations of *γ*-aminobutyric acid and glutamate in patients with major depression. *Archives of General Psychiatry*.

[14] Sheehan D (2000). *Physical Biochemistry: Principles and Applications*.

[15] Lakowicz JR (1990). *Principles of Fluorescence Spectroscopy*.

[16] Natale P, den Blaauwen T, van der Does C, Driessen AJM (2005). Conformational state of the SecYEG-bound SecA probed by single tryptophan fluorescence spectroscopy. *Biochemistry*.

[17] Tyagi NK, Goyal P, Kumar A, Pandey D, Siess W, Kinne RKH (2005). High-yield functional expression of human sodium/D-glucose cotransporter1
in *Pichia pastoris* and characterization of ligand-induced conformational changes as studied by tryptophan fluorescence. *Biochemistry*.

[18] Amsterdam J, Brunswick D, Mendels J (1980). The clinical application of tricyclic antidepressant pharmacokinetics and plasma levels. *American Journal of Psychiatry*.

[19] Baldessarini RJ, Gilman AG, Goodman LS (1980). Drugs and the treatment of psychiatric disorders. *The Pharmacological Basis of Therapeutics*.

[20] Kuusinen A, Arvola M, Keinänen K (1995). Molecular dissection of the agonist binding site of an AMPA receptor. *The EMBO Journal*.

[21] Ivanovic A, Reiländer H, Laube B, Kuhse J (1998). Expression and initial characterization of a soluble glycine binding domain of the *N*-methyl-D-aspartate receptor NR1 subunit. *Journal of Biological Chemistry*.

[22] Keinänen K, Jouppila A, Kuusinen A (1998). Characterization of the kainate-binding domain of the glutamate receptor GluR-6 subunit. *Biochemical Journal*.

